# Critical Roles of Impurities and Imperfections in Various Phases of Materials

**DOI:** 10.3390/ma16041612

**Published:** 2023-02-15

**Authors:** Kyuichi Yasui

**Affiliations:** National Institute of Advanced Industrial Science and Technology (AIST), Nagoya 463-8560, Japan; k.yasui@aist.go.jp

**Keywords:** impurities, bulk nanobubble, cavitation threshold, strength of brittle ceramics, pre-existing microcracks, size effect of a BaTiO_3_ nanocube, adsorbate, defects, dislocations, flexoelectric polarization

## Abstract

In many materials, impurities and imperfections play a critical role on the physical and chemical properties. In the present review, some examples of such materials are discussed. A bulk nanobubble (an ultrafine bubble) is stabilized against dissolution by hydrophobic impurities attached to the bubble surface. An acoustic cavitation threshold in various liquids decreases significantly by the presence of impurities such as solid particles, etc. The strength of brittle ceramics is determined by the size and number of pre-existing microcracks (imperfections) in the specimen. The size effect of a BaTiO_3_ nanocrystal is influenced by the amount and species of adsorbates (impurities) on its surface as adsorbate-induced charge-screening changes the free energy. The dielectric constant of an assembly of BaTiO_3_ nanocubes is influenced by a small tilt angle (imperfection) between two attached nanocubes, which induces strain inside a nanocube, and is also influenced by the spatial strain–relaxation due to defects and dislocations (imperfections), resulting in flexoelectric polarization.

## 1. Introduction

In first-principles calculations—such as density functional calculations for quantum mechanics, the finite element method (FEM) applied to mechanics of materials, computational fluid dynamics (CFD), and molecular dynamics simulations—the effects of impurities and imperfections are sometimes neglected [1,2,3,4,5,6,7,8,9,10,11,12,13]. On the other hand, it has been discussed that most of the material properties of crystalline solids are governed by imperfections (defects) such as diffusion, strength and plasticity, dielectric permittivity, electrical conductivity, etc. [14]. As the solid-state sintering of ceramics or metals is strongly influenced by the bulk diffusion coefficient, the densification rate in solid-state sintering is strongly influenced by defect concentration [15,16,17,18]. Nonstoichiometry in ionic crystals is a kind of imperfection and results in a higher density of defects and higher diffusion coefficient [15,19]. The crystal habit is also modified by the presence of impurities [20]. Not only for crystalline solids but also for other kinds of physical, chemical, and electrical systems, impurities and imperfections sometimes play important roles. In the present review, some examples of critical roles of impurities and imperfections in various phases of materials are discussed in order to point out the importance of impurities and imperfections. Here, various phases of materials mean the gas, liquid, and solid phases of materials. In the next section, the impact of impurities on the stability of a bulk nanobubble, which is also called an ultrafine bubble, is discussed. A bulk nanobubble is a gas bubble smaller than 1 μm in diameter [21,22]. Bulk nanobubbles have been commercially applied to cleaning, washing machines, plant cultivation, etc. [21]. Although numerous papers [23,24,25,26,27,28,29] have been published on the stabilization mechanism of a bulk nanobubble by electrostatic pressure caused by the charged surface of a bulk nanobubble, the TEM observation shown in the next section indicates that a bulk nanobubble is stabilized against dissolution by being partly covered by hydrophobic impurities [30].

## 2. Bulk Nanobubbles (Ultrafine Bubbles)

Bulk nanobubbles have been experimentally studied since around 2000 [21,22]. The term “bulk” nanobubble means a nanobubble in bulk liquid and is used to distinguish from surface nanobubbles, which are gas objects on a solid surface [22,31,32,33]. According to the ISO standardization [34], the terminology ultrafine bubble (UFB) is used instead of bulk nanobubble in the following. The definition of UFBs is the same as that of bulk nanobubbles, which does not include microbubbles [34]. The production of UFBs is usually by hydrodynamic or acoustic cavitation [35]. During hydrodynamic cavitation to produce UFBs, the liquid water becomes “milky” due to the generation of numerous microbubbles [36]. After stopping hydrodynamic cavitation, most of the microbubbles gradually move upward by buoyancy and disappear at the liquid surface [37]. After a few minutes, the liquid returns to being transparent. In the transparent liquid water, however, there are many UFBs, which can be confirmed by particle tracking analysis or simply by the Tyndall effect using a laser pointer [35,38,39]. In the experiment of Kanematsu et al. [40], for example, UFBs had diameters ranging from 40 to 600 nm. The number density of UFBs larger than 400 nm was very low, and the typical diameter was 70 nm [32,40]. Surprisingly, the typical lifetime of UFBs is more than 200 days in spite of the fact that the Epstein–Plesset theory of bubble dissolution predicts the typical lifetime to be less than 0.1 ms [40,41,42,43,44]. There is a criticism that the observed UFBs are not gas bubbles but solid or liquid impurities [45,46]. However, Kanematsu et al. [40] experimentally reported that over 90% of the particles disappeared after the freeze–thaw process, which suggests that most of the particles are gas bubbles. Other research groups have also experimentally reported the disappearance of the particles after the freeze–thaw process [47,48]. There are also many other pieces of evidence that indicate most of the particles are gas bubbles [47,49,50,51,52,53].

**Figure 1:**
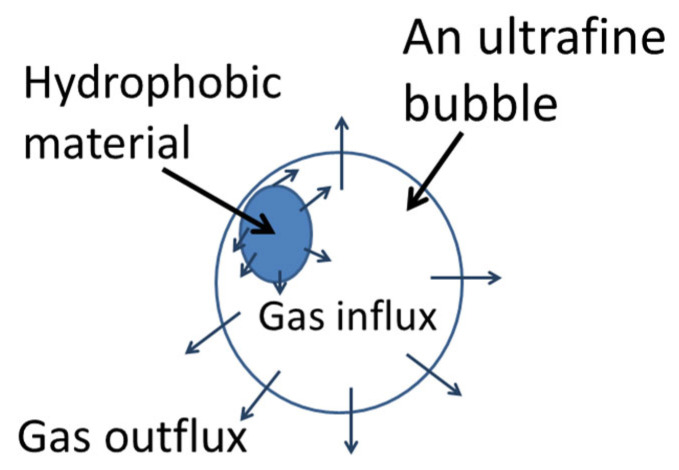
Dynamic equilibrium model of an ultrafine bubble (bulk nanobubble) partly covered with hydrophobic material (impurity). Reprinted with permission from Ref. [56]. Copyright 2016, the American Chemical Society.

**Figure 2:**
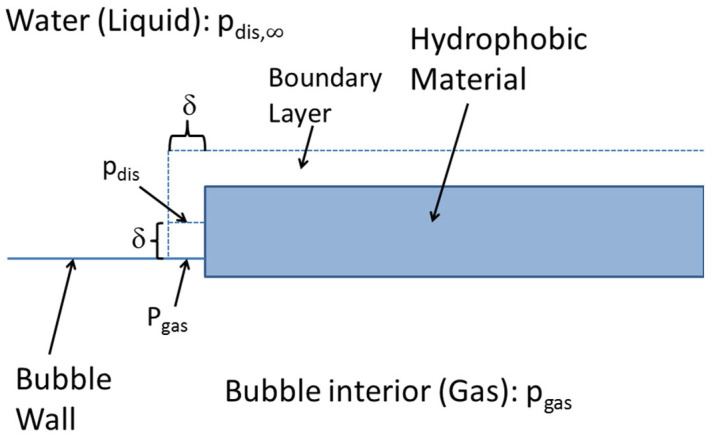
Enlarged view at around the bubble wall partly covered with hydrophobic material (impurity). Reprinted with permission from Ref. [56]. Copyright 2016, the American Chemical Society.

There are various theoretical models for the stability of UFBs [41,54,55]. In the author’s opinion, the most promising model is the dynamic equilibrium model because there is experimental evidence of images, and the reduction in surface tension of UFB water is solely explained by the model [49,55,56,57,58,59,60,61]. The dynamic equilibrium model is as follows [56]: A gas bubble is assumed to be partly covered with hydrophobic impurities such as oils, carbon particles, etc., as shown in Figure 1 [56]. As a hydrophobic material repels water, there is a thin density depletion layer on the surface of a hydrophobic material [62,63]. The thickness of a density depletion layer ranges from 0.2 to 5 nm [62,63]. The density of water in the depletion layer is from 44 to 94% of that of normal water [62,63]. In the depletion layer, gas dissolved in water is preferentially trapped [64,65,66,67,68]. The gas pressure in the depletion layer is as high as about 67 atm when the hydrophobic potential is $1.7\times 10^{-20}$ J at 20 °C in gas-saturated water [33,49,69,70]. Now, gas influx near the periphery of a hydrophobic material attached to a bubble surface is discussed (Figure 2) [56]. The gas pressure in liquid water at the bubble wall is roughly equal to the internal gas pressure, which is about 15 atm according to the Laplace pressure, due to surface tension [41,42,71]. In the depletion layer (the boundary layer in Figure 2), apart from the bubble surface, the gas pressure in liquid water is as high as about 67 atm, as discussed above. Accordingly, there is a strong gradient of gas concentration in liquid water along the boundary layer in Figure 2 near the bubble wall. Due to the gradient of gas concentration, gas diffuses into the bubble near the periphery of the hydrophobic material attached to a bubble surface.

On the other hand, at the uncovered surface of a bubble, gas diffuses out of a bubble because the internal gas pressure (15 atm) is much higher than the ambient gas pressure (1 atm) in the liquid water. When the gas influx and outflux are balanced, gas dissolution is stopped. In addition, the balance of gas influx and outflux should be in stable equilibrium such that a slight change in bubble radius results in the return to the equilibrium radius. Numerical calculations of these conditions have revealed that a UFB can be stable when more than 50% of the bubble surface is covered with hydrophobic materials [56].

**Figure 3:**
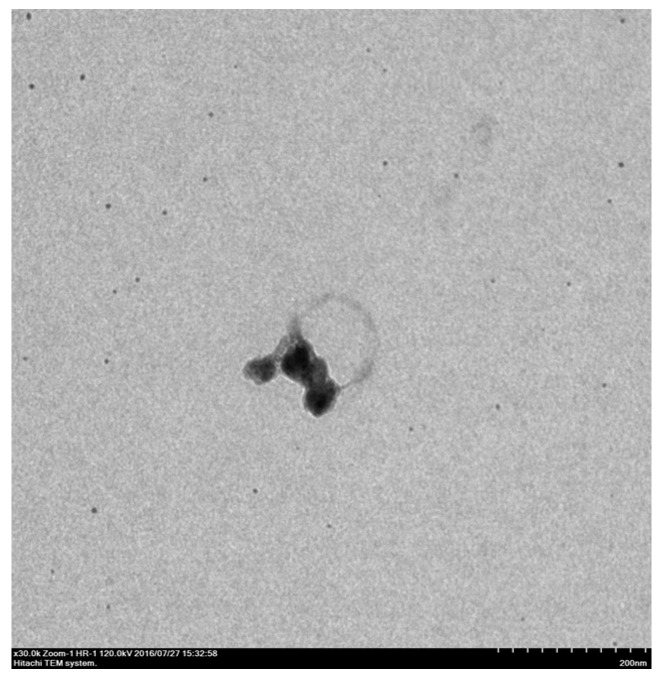
The TEM image of a UFB partly covered with a hydrophobic impurity (oleic acid). The diameter of the UFB is 105 nm. Reprinted with permission from Ref. [30]. Copyright 2017, the Japanese Society for Multiphase Flow.

Sugano, Miyoshi, and Inazato [30,57] experimentally reported the TEM images of stable UFBs partly covered with hydrophobic materials in aqueous solutions without freezing. An example of the TEM images of UFBs is shown in Figure 3 [30]. This is a piece of evidence for the dynamic equilibrium model. Furthermore, as more than 50% of the bubble surface is covered with hydrophobic materials according to the dynamic equilibrium model, UFBs can be stable at the liquid surface when the covered part of the bubble surface is directed toward the gas phase [58,60]. On the other hand, uncovered bubbles burst at the liquid surface and immediately disappear. Accordingly, any models of uncovered bubbles such as the electrostatic-charge stabilization model [23,24,25,26,27,28,29] fail to explain the reduction in surface tension of UFB water. In summary, UFBs are stabilized against dissolution by being partly covered with hydrophobic impurities. In other words, impurities play an essential role in stabilizing UFBs. There is a question as to whether impurities truly exist in deionized water, in which stable UFBs are observed [54]. The answer is that impurities could be produced from a “UFB generator”.

## 3. Acoustic Cavitation Threshold

When liquid is irradiated with strong ultrasound, numerous gas bubbles are formed, and they repeat expansion and collapse with the acoustic period, which is called acoustic cavitation [71,72,73]. Ultrasound is a propagation of pressure oscillation with a sound speed with higher frequency than 20 kHz (or 10 kHz), which is inaudible [71,72,74,75]. Under acoustic cavitation, many bubbles expand during the rarefaction phase of ultrasound and collapse violently at the compression phase of ultrasound. At the end of violent collapse, the temperature and pressure inside a bubble increase to more than 4000 K and 300 bar, respectively [76,77]. As a result, water vapor and oxygen (if present) are dissociated inside a heated bubble, and oxidants such as OH and O radicals are produced, which is called sonochemical reactions [71,72,73,74,78,79]. Furthermore, a faint light is emitted from a heated bubble in which gases are weakly ionized, partly due to the ionization potential lowering as a result of the high density inside a heated bubble, which is called sonoluminescence (SL) [80,81,82,83]. It has been experimentally reported that SL intensity increases by the presence of UFBs in water because UFBs work as cavitation nuclei [84,85].

The tensile strength of pure water is theoretically calculated from the work required to create a bubble or from a van der Waals equation of state for a liquid as of the order of 1000 atm [86,87]. The minimum pressure–amplitude of ultrasound for cavitation to occur is called the cavitation threshold. The experimentally determined cavitation threshold is more than one order of magnitude lower than the theoretical tensile strength of pure water [42,88]. The experimentally determined cavitation threshold decreases as the gas concentration in liquid water increases [88]. In air-saturated water, the cavitation threshold is only about 1 atm [88]. The reason for the huge discrepancy between the experimentally determined cavitation threshold and the theoretical tensile strength of pure water is the presence of impurities in liquid in actual experiments, which are called cavitation nuclei [71,72,73]. There are mainly two types in cavitation nuclei (impurities): One is solid particles, and the other is tiny gas bubbles including UFBs [71,72,73,89,90,91,92,93,94].

**Figure 4:**
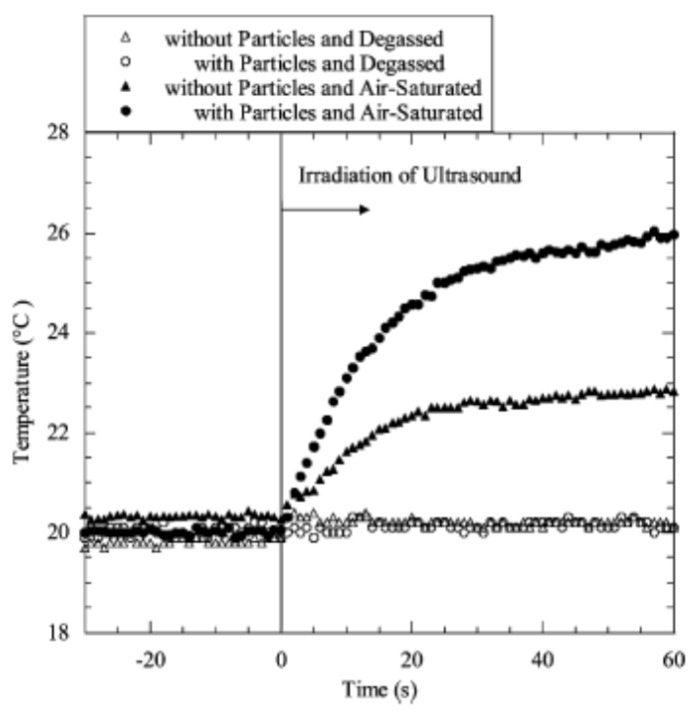
Liquid temperature as a function of time with and without alumina particles of 10 μm in diameter in air-saturated or degassed water irradiated with ultrasound of 42 kHz. Reprinted with permission from Ref. [96]. Copyright 2005, the American Chemical Society.

Bubbles are easily nucleated from a crevice on a solid particle. The mechanism for the nucleation of bubbles from a crevice on a solid particle is explained in the literature [42,71,72,73,89,95]. Tuziuti et al. [96] showed experimentally that the addition of an appropriate amount of alumina particles of appropriate size (10 μm) enhanced the acoustic cavitation, which was measured by a temperature increase of the liquid water (Figure 4). As the source of the heat is cavitation bubbles, the liquid temperature increases more when the number of cavitation bubbles is larger [97]. Hydrophobic particles work more effectively as cavitation nuclei compared to hydrophilic particles [98,99]. In summary, the number, size, and hydrophobicity of impurity particles determine the acoustic cavitation threshold (or number of cavitation bubbles) [100].

## 4. Strength of Brittle Ceramics

A material fractures when sufficient stress is applied on the atomic level to break the atomic bonds [101]. Theoretical estimate of the cohesive strength at the atomic level is approximately E/π, where E is the Young’s modulus of the material [101]. However, the experimental fracture strengths for brittle materials are typically 3 or 4 orders of magnitude below this value [101]. The discrepancy between the actual strengths of brittle materials and the theoretical estimates is due to pre-existing microcracks (imperfections) in these materials. Due to the presence of a microcrack, local stress is magnified at the tip of a microcrack, which strongly lowers the global strength of the material [101]. In other words, local stress at the tip of a microcrack can exceed the theoretical cohesive strength under a significantly lower global applied stress [101]. Griffith [102] treated the problem by considering the energy balance. For the increase in the crack area (for crack propagation), the potential energy supplied by the internal strain energy and external stresses should exceed the work required to create new surfaces [101]. The tensile strength ($\sigma_{t}$) of a material is obtained by the balance between the potential energy and the surface energy as follows for a penny-shaped microcrack embedded in the material [101].

(1) $$\sigma_{t}=\sqrt{\frac{\pi E\gamma_{s}}{\left(1-\nu^{2}\right)d}}$$

where $\gamma_{s}$ is the surface energy of the material per unit area, ν is Poisson’s ratio, and d is the crack diameter. The surface energy ($\gamma_{s})\;$ of crystalline ceramics ranges from about 0.5 J m^−2^ to about 3 J m^−2^ [103,104]. Young’s modulus (E) of ceramics ranges from about 20 GPa to about 570 GPa [105,106]. Poisson’s ratio (ν) of ceramics ranges from about 0.1 to 0.3 [105]. Accordingly, for a typical diameter (d) of a microcrack of 1–10 μm which is in the same order of magnitude as that of the grain size [107,108], the Griffith tensile strength given by Equation (1) yields about 3 orders of magnitude smaller value than the theoretical cohesive strength (E/π), which nearly agrees with the experimental data. In the Griffith tensile strength, however, the effect of microcrack formation by the pileup of dislocations at grain boundaries is neglected [101,109,110]. Furthermore, in relatively ductile ceramics, microcrack formation by the pileup of dislocations also occurs at the intersections of dislocation bands (slip bands), and the deviation from the Griffith criterion becomes considerable [111,112,113,114,115,116].

For compressive strength ($\sigma_{c}$), the following relationship crudely holds [117].

(2) $$\sigma_{c}=R\left|\sigma_{t}\right|=8\frac{\sigma_{c}}{\sigma_{ci}}\left|\sigma_{t}\right|$$

where R is the strength ratio, $\sigma_{ci}$ is the crack initiation stress, and $\sigma_{c}$ is the peak strength. The strength ratio (R) ranges from 2 to 64, depending on the material species as well as the specific specimen [117,118].

**Figure 5:**
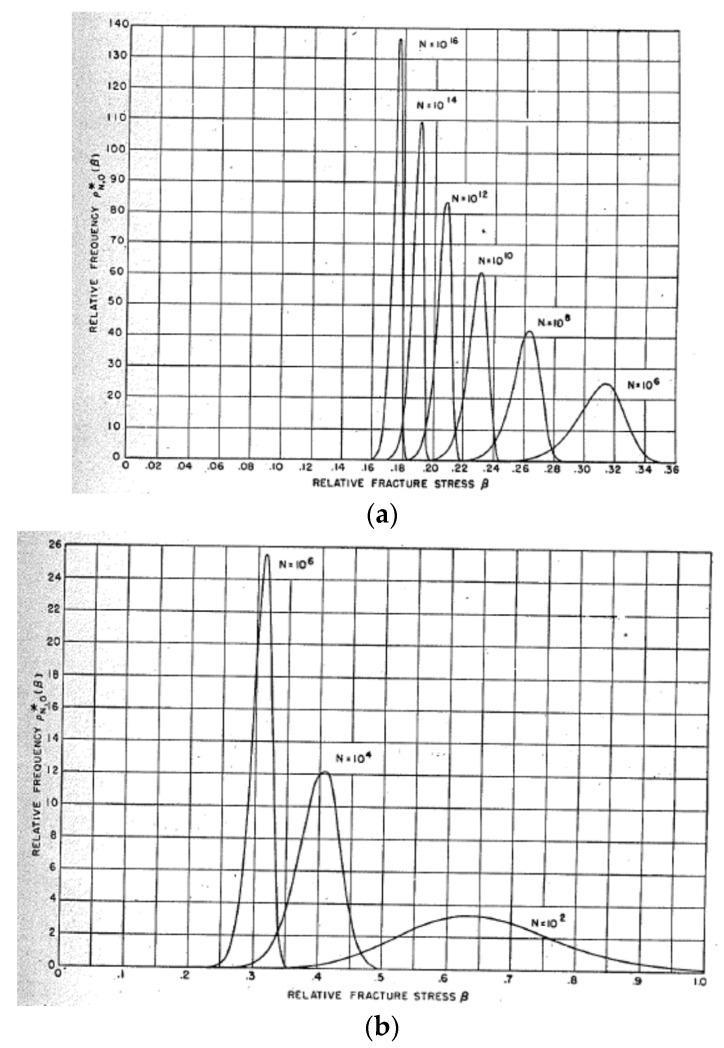
Theoretically predicted frequency of fracture stresses for specimens containing N microcracks. (**a**) The range of N is from 10^6^ to 10^16^. (**b**) The range of N is from 10^2^ to 10^6^. Reprinted with permission from Ref. [119]. Copyright 1947, the Minerals, Metals & Materials Society.

Fisher and Hollomon [119] performed theoretical calculations on the frequency distribution of fracture stresses for specimens with various numbers of pre-existing microcracks under a certain distribution of microcrack diameters (Figure 5). It has been shown that the strength of a specimen depends on the total number (N) of pre-existing microcracks in the specimen. For a small number of pre-existing microcracks, the size of a specimen strongly influences the strength of a specimen because a reduction in N (for example, 100-fold decrease) results in a considerable increase in relative fracture stress for a relatively small N, which qualitatively agrees with the experimental results (Figure 5b) [101,119]. For a large value of N, on the other hand, there is a negligible size effect of a specimen (Figure 5a). It is also predicted that there is a scatter in fracture stress measurements for different specimens of a single material, which qualitatively agrees with the experimental results [101,119]. In summary, the strength of a brittle specimen is mainly determined by the size (diameter) and number of pre-existing microcracks (imperfections) in the specimen.

## 5. Size Effect of a BaTiO_3_ Nanocrystal

**Figure 6:**
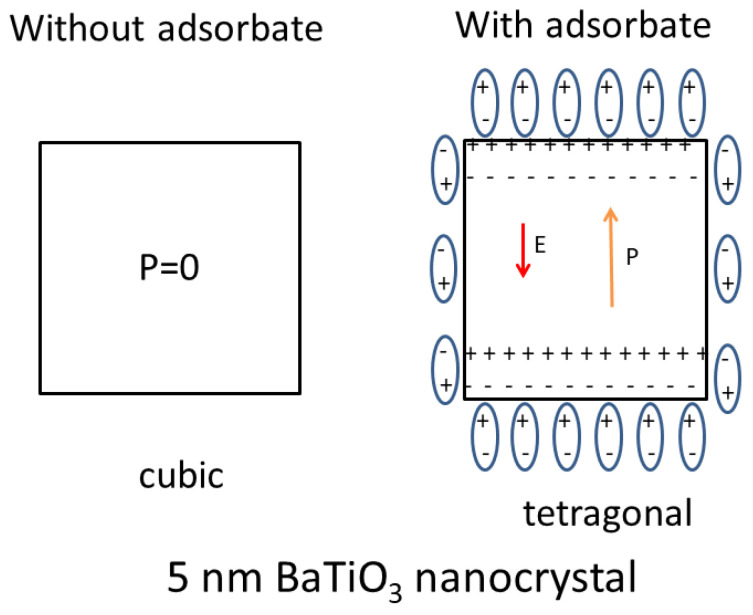
Size effect of a 5 nm BaTiO_3_ nanocrystal with and without adsorbate-induced charge screening. Reprinted with permission from Ref. [131]. Copyright 2013, the American Chemical Society.

Barium titanate (BaTiO_3_) is one of the most important electronic ceramic materials [120]. It has a high dielectric constant at room temperature and is widely used as a dielectric material for ceramic capacitors. Over three trillion BaTiO_3_-based multilayer ceramic capacitors (MLCCs) are used each year [121]. At room temperature, the crystal structure of a bulk BaTiO_3_ single crystal is tetragonal, and it has spontaneous polarization of about 0.15 C m^−2^ [122]. It becomes cubic above the Curie temperature (120 °C), and the spontaneous polarization disappears. There are numerous experimental reports that a BaTiO_3_ single crystal becomes cubic at room temperature when the crystal size is smaller than a critical one, which is called the size effect [123,124,125,126,127,128,129]. Surprisingly, the critical size is largely different for different experiments and ranges from about 5 to 200 nm [123,124,125,126,127,128,129,130]. As is briefly discussed in the followings, the large diversity in the critical size is mostly due to adsorbates (impurities on the surface of a crystal) which partly screen the surface charges and reduce the free energy of tetragonal phase (Figure 6) [131].

Stable crystal structure under a given temperature is determined by the lowest free energy at the temperature among the several crystal structures [132,133]. The size effect of a BaTiO_3_ single crystal is caused by the depolarization energy, which is the positive energy due to depolarization field [131]. When there is spontaneous polarization, some electric charges appear on the surface of a single crystal. The surface charges induce the electric field inside a single crystal in the opposite direction to the spontaneous polarization, which is called the depolarization field (the right of Figure 6) [131,134,135]. The depolarization energy is the positive energy of a dielectric material under an electric field (depolarization field) [134,136]. The actual BaTiO_3_ crystals are not ideal insulators, and there are mobile charge carriers in the crystals [122,133,137]. Accordingly, the mobile charge carriers screen the depolarization field, and the depolarization field exists only near each surface of a BaTiO_3_ single crystal [131,137]. Thus, the depolarization energy density, which is defined by the depolarization energy divided by the volume of the particle (crystal), becomes negligible for a macroscopic crystal. In other words, the depolarization energy density becomes important only for relatively small crystals, which destabilizes the tetragonal crystal structure with spontaneous polarization by increasing the free energy [131,137]. This is the reason for the size effect of a BaTiO_3_ single crystal.

When there is some adsorbate (impurity) on the surface of a BaTiO_3_ single crystal and the surface charge is considerably screened by the adsorbate, the depolarization energy density is considerably decreased because the depolarization energy density is proportional to the square of the polarization at the surface, which could be decreased by the adsorbate-induced charge screening [131]. In other words, adsorbate-induced charge screening weakens the size effect of a BaTiO_3_ single crystal. It has been reported both experimentally and theoretically that molecular adsorbates such as H_2_O, OH, CO_2_, and oleate groups on the surface of BaTiO_3_ single crystals screen the surface charges and influence the stability of the electric polarization of BaTiO_3_ crystals [138,139,140,141,142,143,144,145,146]. In different experiments, the degree of adsorbate-induced charge screening is different because the amount and species of adsorbates are different. This is one of the reasons for the large diversity in the experimental results on the size effect of a BaTiO_3_ single crystal [131].

**Figure 7:**
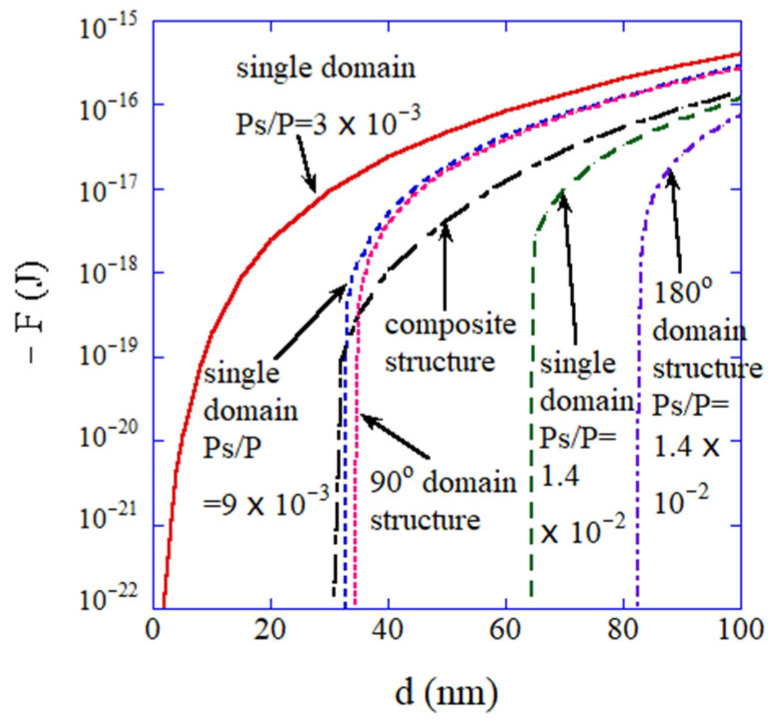
Theoretically calculated free energy as a function of the particle size. Reprinted with permission from Ref. [131]. Copyright 2013, the American Chemical Society.

Some researchers have suggested that some BaTiO_3_ nanoparticles exhibit a composite structure; the crystal structure of a surface layer is cubic, and that of an inner core is tetragonal [147,148,149,150]. There is also the intermediate layer between the inner core and the surface layer, which is called the gradient–lattice–strain layer (GLSL) [148,149,150]. Furthermore, it may be possible that there is a domain structure with 90° or 180° domain walls in a BaTiO_3_ nanoparticle [131,137]. The numerically calculated free energies of a single domain with various degrees of adsorbate-induced charge screening as well as those of the composite structure and the domain structure with 90° or 180° domain walls are shown in Figure 7 for comparison [131]. In Figure 7, $P_{s}/P$ indicates the degree of adsorbate-induced charge screening, where $P_{s}$ is the polarization at the surface after adsorbate-induced charge screening, and P is the polarization inside a BaTiO_3_ nanoparticle. For a relatively small value of $\frac{P_{s}}{P}=3\times 10^{-3}$ which corresponds to a relatively high degree of adsorbate-induced charge screening, the free energy of a single domain is lower ((−F) is larger) than those of a composite structure and the domain structures (Figure 7) [131]. On the other hand, for a smaller degree of adsorbate-induced charge screening ($\frac{P_{s}}{P}=1.4\times 10^{-2}$), the free energy of the domain structure with 90° domain walls as well as that of the composite structure are smaller than that of a single domain—at least for relatively small particles.

In summary, the size effect of a BaTiO_3_ nanoparticle as well as the appearance of the composite structure and the domain structure is strongly influenced by the surface charge screening by adsorbates (impurities on the surface of a nanoparticle).

## 6. Dielectric Constant of an Assembly of BaTiO_3_ Nanocubes

An ordered assembly of BaTiO_3_ nanocubes (nanocrystals) is a candidate for the miniaturization of dielectric devices such as multilayer ceramic capacitors (MLCCs) and positive temperature coefficient thermistors [151,152,153,154]. Mimura and Kato [155,156] have fabricated ordered assemblies of BaTiO_3_ nanocubes (15 nm) capped with oleic acid by the dip-coating method in a mesitylene solution. In dip-coating, the oriented attachment of BaTiO_3_ nanocubes is due to electric dipole–dipole interaction according to the numerical simulations of collisions of two BaTiO_3_ nanocubes [157]. A film of self-assembled BaTiO_3_ nanocubes was calcinated at 400 °C for 1 h and sintered at 850 °C for 1 h in O_2_ [155,156]. There was no observable change in the structure of the ordered assembly even after calcination and sintering, except for the formation of the joint at the crystal interfaces at the atomic level [155,156,158]. Surprisingly, the measured dielectric constant of the film was as high as about 3800 and 2600 for 290- and 580-nm-thick film, respectively, at 1 MHz and room temperature [155,156]. For both thicknesses, the amplitude of the applied alternating electric field in the measurements was 0.5 V. Accordingly, the amplitude of the electric field was larger for a thinner film; 17.24 kV cm^−1^ and 8.62 kV cm^−1^ for 290 nm and 580 nm thickness, respectively. The dielectric constants of the films of BaTiO_3_ nanocube assemblies are much higher than the normal dielectric constant of a BaTiO_3_ bulk crystal (about 1600) without any domain contribution [159,160] and those of typical BaTiO_3_ thin films (lower than 1000) [161,162,163,164,165]. Furthermore, the temperature dependence of the capacitance (dielectric constant) of the film of BaTiO_3_-nanocube ordered assembly was nearly flat with a very broad peak at around 100 °C [155].

It has been suggested that the observed high dielectric constants are explained by flexoelectric polarization inside BaTiO_3_ nanocubes [166,167]. Flexoelectric polarization is electric polarization induced by strain gradient in dielectric crystals regardless of the crystal symmetry [168,169,170,171,172]. The magnitude of flexoelectric polarization is proportional to the strain gradient. The flexoelectric coefficient, which is the coefficient of the proportionality, for BaTiO_3_ is as high as about 10 μC m^−1^ at room temperature [171]. Flexoelectric polarization may be important for thin films and nanomaterials because a strain gradient could be as high as $10^{5}\sim 10^{6}$ m^−1^, which is six to seven orders of magnitude larger than that in bulk solids [168,173,174].

**Figure 8:**
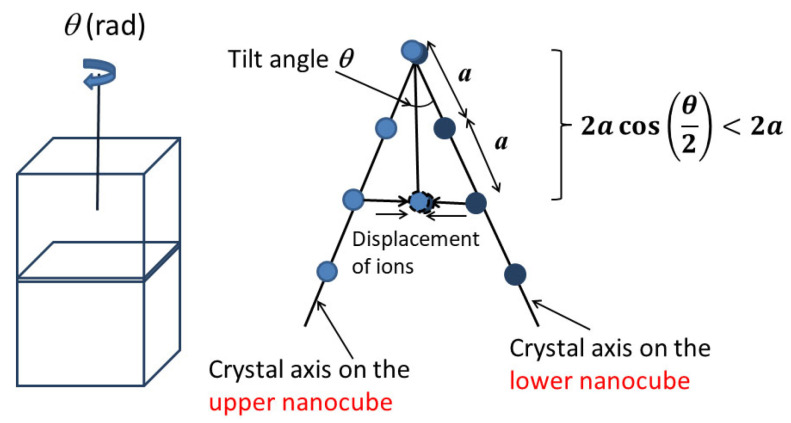
Misfit strain (the right figure) caused by a small tilt angle *θ* between two attached BaTiO_3_ nanocubes (the left figure). The right figure is the projection view of the interface from the above. Two corresponding crystal axes on upper and lower nanocube are shown in the figure. The small circles are Ba ions. Reprinted with permission from Ref. [166]. Copyright 2020, IOP Publishing.

For BaTiO_3_ nanocube assemblies, the strain could be induced by a small tilt angle (imperfection) between two attached nanocubes (Figure 8) [166]. The corresponding crystal axes are aligned by attractive force and the distance between neighboring ions becomes shorter, which implies that compressive strain appears (the right of Figure 8) [166]. The compressive strain ($u_{m}$) is approximately expressed as follows [166].

(3) $$u_{m}=\cos\left(\frac{\theta }{2}\right)-1\leq 0$$

where θ is the tilt angle, and the negative value of $u_{m}$ means the compressive strain.

Due to the presence of defects and dislocations (imperfections), the magnitude of compressive strain decreases as the distance from the interface increases toward the center of a nanocube [166,167]. This means that a strain gradient appears that induces flexoelectric polarization. The magnitude of the flexoelectric polarization (P) is estimated as follows [166,167].

(4) $$P=\mu \frac{\partial \epsilon }{\partial x}\approx -\frac{\mu \cdot u_{m}}{\delta }\approx 2.67\;C\;m^{-2}$$

where μ is the flexoelectric coefficient (10 μC m^−1^), ∂ϵ/∂x is the strain gradient, δ is the width of the strain region (δ≈d/2 is assumed, where d is the size of a nanocube (15 nm)), and θ=7.2° is assumed in Equation (3) because the tilt angle θ was experimentally estimated to be less than 10° [158]. Thus, the estimated magnitude of flexoelectric polarization is about one order of magnitude larger than the spontaneous polarization of BaTiO_3_.

**Figure 9:**
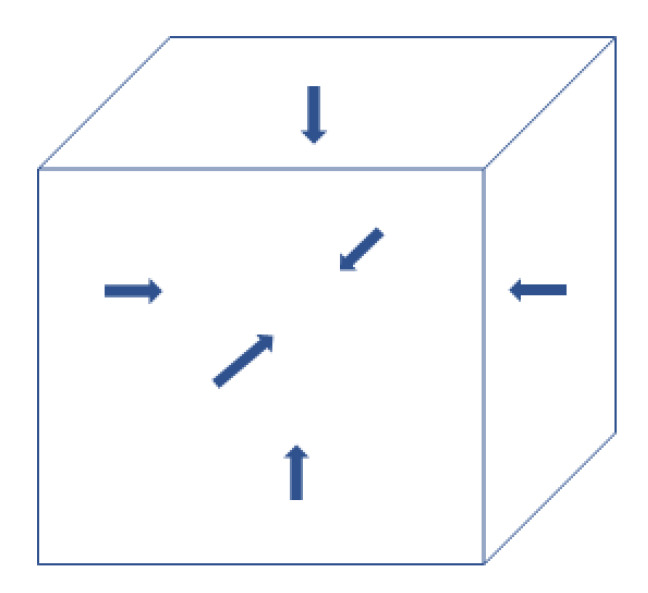
Flexoelectric polarization near each surface of a BaTiO_3_ nanocube. At each surface of a nanocube, another nanocube is attached in an ordered assembly. Reprinted with permission from Ref. [166]. Copyright 2020, IOP Publishing.

**Figure 10:**
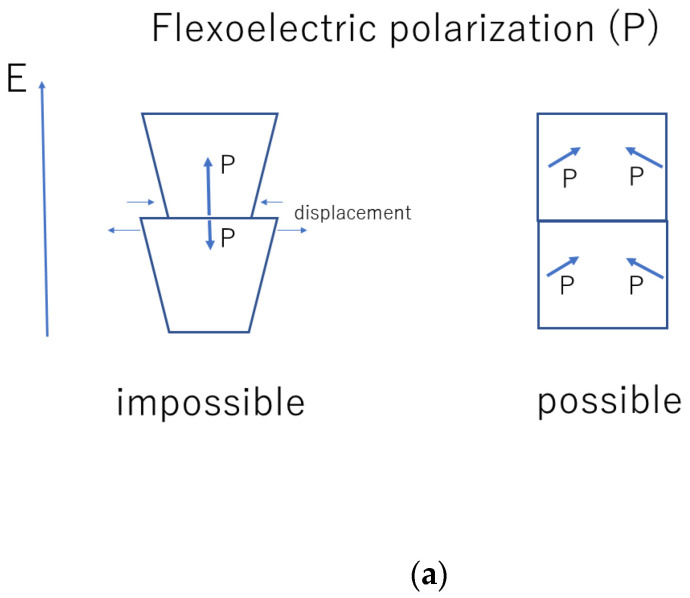

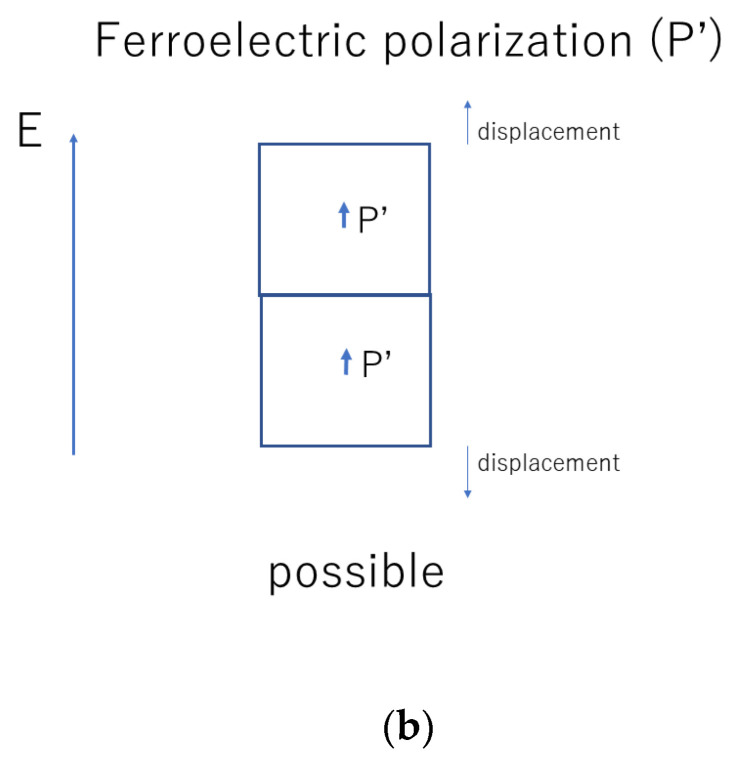
Flexo- (**a**) and ferro-electric (**b**) polarization under applied alternating electric field (E) for tightly joined BaTiO_3_ nanocubes in an ordered assembly. Reprinted with permission from Ref. [167]. Copyright 2020, MDPI.

Accordingly, there are six vectors of flexoelectric polarization inside a nanocube because there are six interfaces (Figure 9) [166]. The flexoelectric polarization parallel to the applied alternating electric field, however, could not respond to the applied electric field because there is a mismatch of strain at the interface as the changes of strain at the interface are different between the two attaching nanocubes (the left of Figure 10a) [167]. In other words, the flexoelectric polarization parallel to the applied alternating electric field does not contribute to the dielectric constant. Instead, the ferroelectric polarization of a BaTiO_3_ nanocube contributes to the dielectric constant (Figure 10b) [167]. On the other hand, the flexoelectric polarization perpendicular to the applied alternating electric field contributes to the dielectric constant (the right of Figure 10a) [167]. Four vectors of the flexoelectric polarization are perpendicular to the applied alternating electric field, and two vectors are parallel to the applied electric field. Accordingly, the dielectric constant (ε) is crudely estimated as follows [167].

(5) $$\varepsilon \approx \frac{2}{3}\varepsilon_{flexo}+\frac{1}{3}\varepsilon_{ferro}$$

where $\varepsilon_{flexo}$ is the dielectric constant due to the flexoelectric polarization perpendicular to the applied electric field, and $\varepsilon_{ferro}$ is the dielectric constant due to the ferroelectric polarization parallel to the applied electric field.

In order to compare with the experimental data of dielectric constant as a function of frequency of applied alternating electric field, the dynamic dielectric-response model of flexoelectric polarization based on ordinary differential equations (ODEs) is used [167]. The model is simply the equation of rotational motion for the electric dipole with the following torques: the torque due to applied alternating electric field, the restoring torque due to anharmonic potential, and the damping torque [167]. Using the model, the dielectric constant due to flexoelectric polarization ($\varepsilon_{flexo}$) is calculated using Equation (6):

(6) $$\varepsilon_{flexo}\approx \frac{{\left(P_{y}\right)}_{amp}}{E_{0}}$$

where $P_{y}$ is the component of polarization parallel to the applied electric field, ${\left(P_{y}\right)}_{amp}$ is the amplitude of temporal variation of $P_{y}$, and $E_{0}$ is the amplitude of the applied alternating electric field. The dielectric constant due to ferroelectric polarization ($\varepsilon_{ferro}$) in Equation (5) is assumed as $\varepsilon_{ferro}\approx 1500$ according to the numerical calculations in Reference [175] and the flat frequency dependence of the dielectric constant for BaTiO_3_ ceramics without domain contribution [159,160].

**Figure 11:**
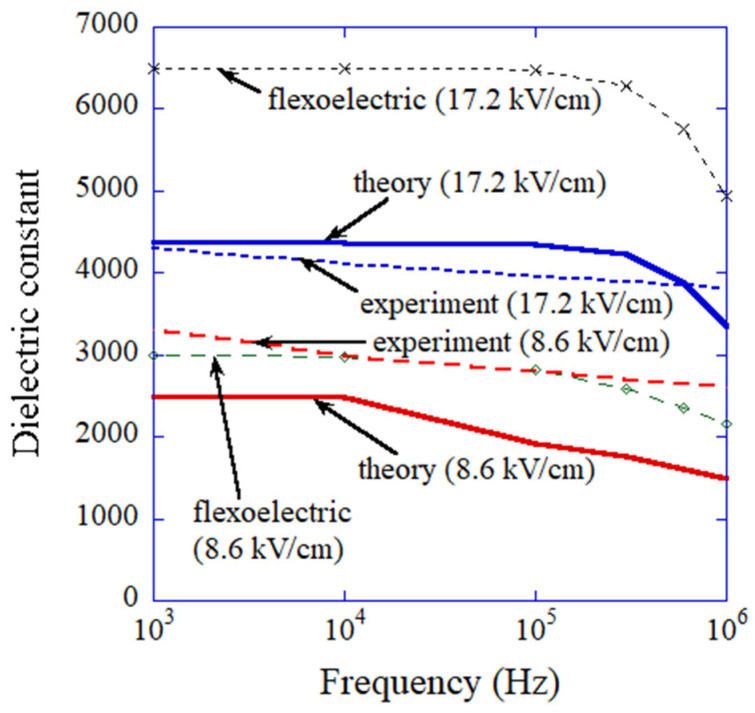
Dielectric constant as a function of frequency. The numerical results on dielectric constant solely by flexoelectric polarization are also shown. The experimental data are also shown for comparison. Reprinted with permission from Ref. [167]. Copyright 2020, MDPI.

The results of the numerical simulations on the frequency dependence of the dielectric constant given by Equation (5) are shown in Figure 11 (labelled with “theory”) with the experimental data for comparison [155,167]. The numerical results of $\varepsilon_{flexo}$ are also shown in Figure 11 (labelled with “flexoelectric”). The results of the numerical simulations (labelled with “theory” in Figure 11) nearly agree with the experimental data, especially for the amplitude of the electric field of 17.2 kV cm^−1^. It suggests that the present model is consistent. However, to validate the ODE model, the existence of flexoelectric polarization in an ordered assembly of BaTiO_3_ nanocubes needs to be confirmed experimentally because the ODE model is not fully based on the first principles [176].

With regard to the nearly flat temperature dependence of dielectric constant of an ordered assembly of BaTiO_3_ nanocubes, the high AC electric field ($E_{0})$ is expected to play an important role because the temperature dependence of the dielectric constant due to ferroelectric polarization becomes nearly flat under high AC electric field [177]. It has already been shown numerically that the temperature dependence of dielectric constant due to flexoelectric polarization is nearly flat [166]. Accordingly, the temperature dependence of the total dielectric constant is expected to be nearly flat, as in the experimental data [155].

Mimura and Kato [178] also fabricated an ordered assembly of barium zirconate titanate (BaZr_x_Ti_(1−x)_O_3_ with x = 0.1 or 0.2) nanocubes (15 nm). The dielectric constant of the assembly after calcination and sintering was as high as about 4000 at 1 MHz at room temperature [178]. Furthermore, the temperature dependence of dielectric constant was nearly flat, as in the case of BaTiO_3_ nanocube assemblies [178]. In other words, dielectric constants of BZ_x_T nanocube assembly and BT (BaTiO_3_) nanocube assembly are similar, as is their temperature dependence. It suggests that there is considerable contribution of flexoelectric polarization on the dielectric constant of an assembly because the dielectric response of flexoelectric polarization does not strongly depend on the species (BZ_x_T or BT) in contrast to that of ferroelectric polarization [177,179,180].

In summary, the high dielectric constant of an ordered assembly of BaTiO_3_ nanocubes as well as its frequency dependence may be explained by flexoelectric polarization induced by strain gradient caused by the small tilt angle (imperfection) between two attached nanocubes and dislocations (imperfection) inside a nanocube.

## 7. Conclusions

The importance of impurities and imperfections in various materials is discussed. Ultrafine bubbles (bulk nanobubbles) are stabilized against dissolution by being partly covered with hydrophobic impurities. The acoustic cavitation threshold as well as the number of cavitation bubbles is determined by the number, size, and hydrophobicity of impurity particles in the liquid. The strength of brittle ceramics is mainly determined by the size and number of pre-existing microcracks (imperfections) in the specimen. The size effect of a BaTiO_3_ nanocrystal is strongly influenced by the surface charge screening by adsorbates (impurities on the surface of a nanocrystal). High dielectric constant of an ordered assembly of BaTiO_3_ nanocubes may be explained by flexoelectric polarization caused by the small tilt angle (imperfection) between two attached nanocubes as well as defects and dislocations (imperfections) inside a nanocube.

## Data Availability

Not applicable.
